# The effect of small-sided games using the FIT LIGHT training system on some harmonic abilities and some basic skills of basketball players

**DOI:** 10.3389/fspor.2023.1080526

**Published:** 2023-01-24

**Authors:** Ahmed K. Hassan, Mohammed S. Alibrahim, Yasser Abdul Rashid Sayed Ahmed

**Affiliations:** ^1^ Department of Physical Education, College of Education, King Faisal University, Al-Ahsa, Saudi Arabia; ^2^ Department of Team Sports and Racket Games, Faculty College of Physical Education, Minia University, Minya, Egypt; ^3^ Department of Curriculum and Instruction, Faculty of Physical Education, Minia University, Minya, Egypt

**Keywords:** small games, basketball, harmonic abilities, fitLight, skills development

## Abstract

**Introduction:**

The aim of this research is to identify the effect of Small-sided games using the FITLIGHT training system on some of the harmonic abilities and some of the basic skills of basketball Players.

**Methods:**

The researchers used the experimental method on 24 basketball players. They were randomly divided into two groups: one experimental (n = 12, age = 10.92 ± 0.79 years; height = 138.50 ± 2.78 cm; weight = 40.25 ± 2.01 kg) and a control group (n = 12, age = 11.17 ± 0. 72 years; length = 139.92 ± 3.53 cm; weight = 40.50 ± . 1.73 kg). The homogeneity between the two groups was calculated and showed that there were no differences between the two samples. In the research variables, the proposed program was applied for 10 weeks at the rate of 4 weekly training units.

**Results and Discussion:**

The proposed training program had a positive impact on the harmonic abilities and basic skills of basketball players and the differences in improvement rates for all variables were in favor of the experimental group. Therefore, the research suggests that Small-sided games using FITLIGHT positively affect all the variables under research. This leads to significant differences between the post-tests and benefits the experimental group.

## 1. Introduction

Basketball has become the third most popular sport in the world (1), being played in almost every nation without exception (2). The world is witnessing a significant and accelerating technological revolution. Competition between countries has become mainly based on the technological capabilities. Therefore, it has been necessary to connect technology and scientific progress. Montella M. et al. (3) show that basketball is a team sport that focuses on the quantitative aspects of performance, strength and endurance. It emphasizes technical and tactical aspects of quality. Basketball is a highly intermittent team sport, with players performing repeated bouts of high-intensity activity interspersed with periods of low to moderate-intensity activity (4, 5). Modern basketball has become characterized by fast rhythm and a high level of skill (6). This is shown by the advanced global level of skill across various tournaments and basketball's engagement with modern trends in training young players (7).

Gabbett T. et al. (8) states that Small-sided games are essential to advance the fields of education and training through the practice of sports activities. They constitute an important part educational and training activities and hold a prestigious position among the various games. Furthermore, they are important in the training of motor, physical, functional and other skills. They have become one of the necessary components within any training program across different age group. Appropriate training curricula based on small games can reach the best developmental results and achieve an advanced level of performance. Games-based drills are an effective training strategy frequently used by basketball coaches (9, 10). In fact, games-based drills represent a major component of the training process in basketball teams as a means to deliver sport-specific physical, physiological, and tactical stimuli in the training environment (9).

Davids K.et al. (11) indicates that Small-sided games are formats of play. The coach adjusts the formats according to the number and abilities of players, the composition of the field, and the specific rules of the game. This presents a specific tactical problem for collective athletic players and improves their abilities and skills. Furthermore, it pro-motes changes in players' tactical behaviors and technical execution because they are playing a modified version of their sport which preserves its basic dynamics and characteristics. In order to reproduce the physical, technical, and tactical require-ments of real match play (12), coaches regularly use small-sided games in their training schedules. Besides their original use for the systematic development of more complex team behaviors for beginners (12), according to the common pedagogical principles from simple to complex and from easy to hard, small-sided games mean while not only became common drills to simultaneously challenge the physical, technical, and tactical qualities in team sports (8, 13).

Small-sided games, known as skills based adapted games or game based training, are used in training players in many sports and in various training methods to improve the functional and athletic skills of players. Small games are widely used by basketball coaches to simultaneously develop technical and tactical skills (12, 14, 15). They also lead to significant improvements in physical and physiological performance under high physical loads (16, 17).

FITLIGHT technology is a training device that is used globally in sports training. Its light stimulus can contribute to improving young basketball players' physical, skill based and visual performances (18). Furthermore, the light stimulus improves their basic skills and increases their ability to perform in various sports. It also develops the physical, harmonic and visual abilities of young players, which leads to responsiveness, agility and compatibility, helping to produce a well-rounded athlete (19). The FITLIGHT training system is versatile and dynamic. It captures various attributes of human performance, such as reaction time, speed and agility. It provides real-time performance feedback and consists of 8 RGB LED powered lamps controlled by a tablet. The lights are used as targets for the user to deactivate and can be adapted and configured for all sports and training systems. This technique is an excellent tool to develop basketball skills and can be used in other sports as well, as it provides athletes and coaches with an advanced training system to develop hand-eye coordination, balance and strength. It can also be used for conditioning and rehabilitation after injury (20).

Glassuer G (21). points out that harmonic ability has a significant and positive role in promoting the speed of learning and mastering motor skills. When a beginner lacks harmonic abilities, he cannot perform the skill correctly and displays many technical errors. A player's possession of harmonic abilities in various sports activities contributes to reducing the acquisition time and promotes the mastery of motor skills, which depend directly on the functional and morphological competence of players.

A basketball player must possess many basic skills. The success of any team and their potential to reach high levels depends on the possession of those skills and their ability to utilize them with a high degree of efficienc. Basic skills are central to basketball. For the player to reach a high skill level, they must possess harmonic abilities and coaches must provide adequate training and utilize advanced methods (18).

Despite the efforts made by scientists, experts and specialists to obtain outstanding results in training and learning methods, the educational and training process still relies on traditional methods in teaching and developing motor skills. Through the experience of researchers and access to previous studies, researchers identified a weakness in the harmonic abilities and basic skills of basketball players. There is a need for new methods which are easy to apply to improve harmonic abilities and the performance of basic basketball skills. There are no previous studies which investigate the development of harmonic abilities or the basic skills of basketball players through Small-sided games. Using FITLIGHT, the study addresses this gap and focuses on harmonic abilities and basic skills from multiple perspectives. The researchers perceived the development of harmonic abilities and basic basketball skills through Small-sided games using FITLIGHT. They recognized that it is a highly engaging training programme which aida harmonic abilities and some basic skills of basketball players. This motivated the researchers to conduct this study. This study is unique as it investigates Small-sided games using FITLIGHT technology. It attempts to highlight the benefits of Small-sided games using FITLIGHT to specialists and trainers. It shows their ability to aid the development of harmonic abilities and performance skills of basketball players. In accordance, This study aims to identify the impact of Small-sided games using FITLIGHT on some of the harmonic abilities and some of the basic skills of basketball players.we hypothesize that: (a) There are statistically significant differences between the average pre- and post-measurements of the experimental group in the harmonic abilities and basic skills of basketball players, and the percentage of improvement favors the telemetry; (b) There are statistically significant differences between the mean pre- and post-measurements of the control group in their harmonic abilities and basic skills, and the percentage of improvement favors the telemetry; and (c) There are statistically significant differences between the mean dimensional measurements of the experimental groups and the control in their harmonic abilities and basic skills, and the percentage of improvement favors the experimental group.

## 2. Materials and methods

### 2.1. Experimental design

The program proposed by the researchers was used for 10 weeks to improve some of the harmonic and skill abilities of young basketball players in Al-Adalah Club, Al-Ahsa, Eastern Province, Kingdom of Saudi Arabia. The training program included small games using FITLIGHT. This program was implemented on an experimental sample of 12 basketball juniors, average in (age = 10.92 ± 0.79 years; height = 138.50 ± 2.78 cm; weight = 40.25 ± 2.01 kg). The research included the existence of a control group of 12 players, training in the traditional way. The comparison of these groups allowed the identification of the effect of Small-sided games using FITLIGHT on some harmonic abilities and some basic skills for basketball juniors. Ethical approval was received from the relevant university committee. The athletes were fully informed of the risks and benefits of the study prior to their entry and they signed an institutionally approved informed consent form. In addition, signed parental consent was obtained for the athletes. The protocol was approved by the Research Ethics Committee at King Faisal University, No., KFU-REC-2022-JAN-ETHICS482.

### 2.2. Tools and devices

**Figure 1:**
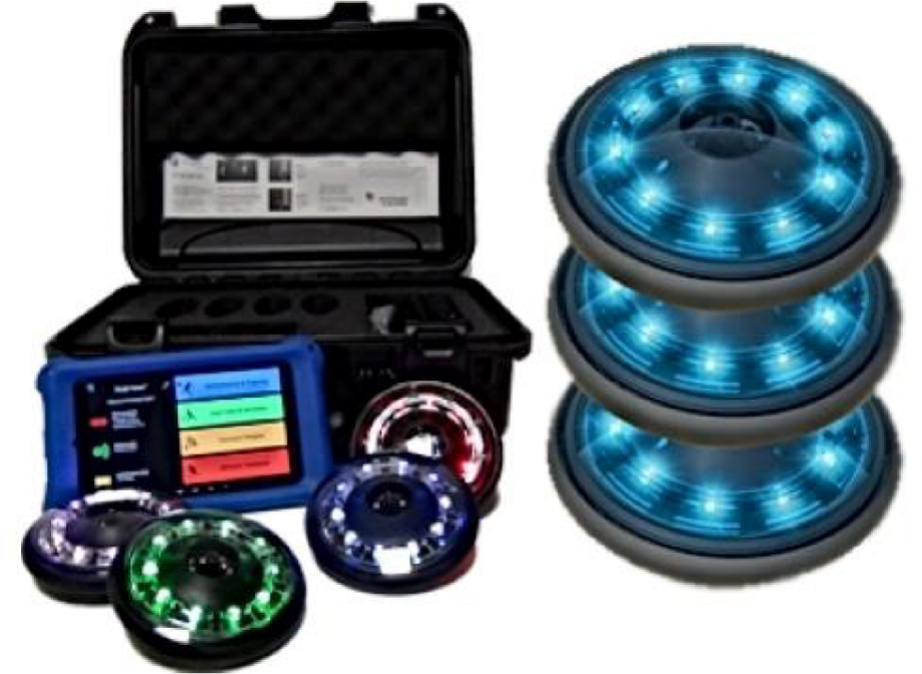
The FIT LIGHT training system of small-sided games with and without basketball.

To collect data for the research, the researchers used A balance for weight measurements, a rastameter for height measurements, a basketball court, a basketball, colored plastic cones for use in the training program exercise app, a stop clock, mattresses, tape measure, a FITLIGHT device In Figure 1, stands for carrying the FITLIGHT disc.The validity and reliability of the tools and devices used were confirmed by comparing the results of some of the devices used by measuring other devices of the same type under the same conditions. These gave the same results, which indicates the reliability and stability of the results. Table 1 shows the weeks, and months of the training program (see Appendixes A–C for more de-tails.)

**Table 1:** The content, techniques used in the sports program.

Weeks	1	2	3	4	5	6	7	8	9	10
Stages	General Preparation Phase			Special Preparation Phase					Competition Preparation Phase	
Pregnancy										
Maximum						•		•		
High		•	•		•				•	
minimum	•			•			•			•
Weekly Time	240 min	320 min	320 min	240 min	320 min	400 min	240 min	400 min	320 min	240 min
Total Time	880 min			1,600 min					560 min	
TotalPrograme Time	3,040 min									

### 2.3. FIT LIGHT training system

#### 2.3.1. Procedures

Procedures to Implement **the Small- Sided Games** Program Using FITLIGHT:

The researchers selected a **Small-sided** games. The duration of the training program with small games was 10 weeks. The number of units per week was 4 and the total number of units was 40. The time of each unit ranged between 60 and 100 min. The **Small-sided** games using FITLIGHT lasted between 20 and 30 min. The experimental group followed the following steps:

- The unit began by giving general warm-up exercises, preparing the various muscles of the body. Special warm-up exercises also took place.
- The researchers explained the **Small-sided** games using FITLIGHT that were applied during the unit, according to the objectives.
- Members of the experimental group performed the small game set using FITLIGHT during the warm-up part and the main part of the unit.

Researchers oversaw the work of the experimental group, provided assistance when needed, and corrected errors if any.

The unit ends with cool-down exercises.

Table 1 shows the time distribution of the training program applied to the experimental group for a period of Ten weeks.

Table 1 shows the time distribution of the training program applied to the experimental group for a period of Ten weeks, where the program was divided into the general preparation stage, lasting three weeks, the special preparation stage, lasting five weeks, and the pre-competition stage, lasting two weeks.

Table 2 shows the protocol for the use of Fitlight within a training session as well as the small-sided game.

**Table 2:** Shows model of a small game using the FIT LIGHT training system.

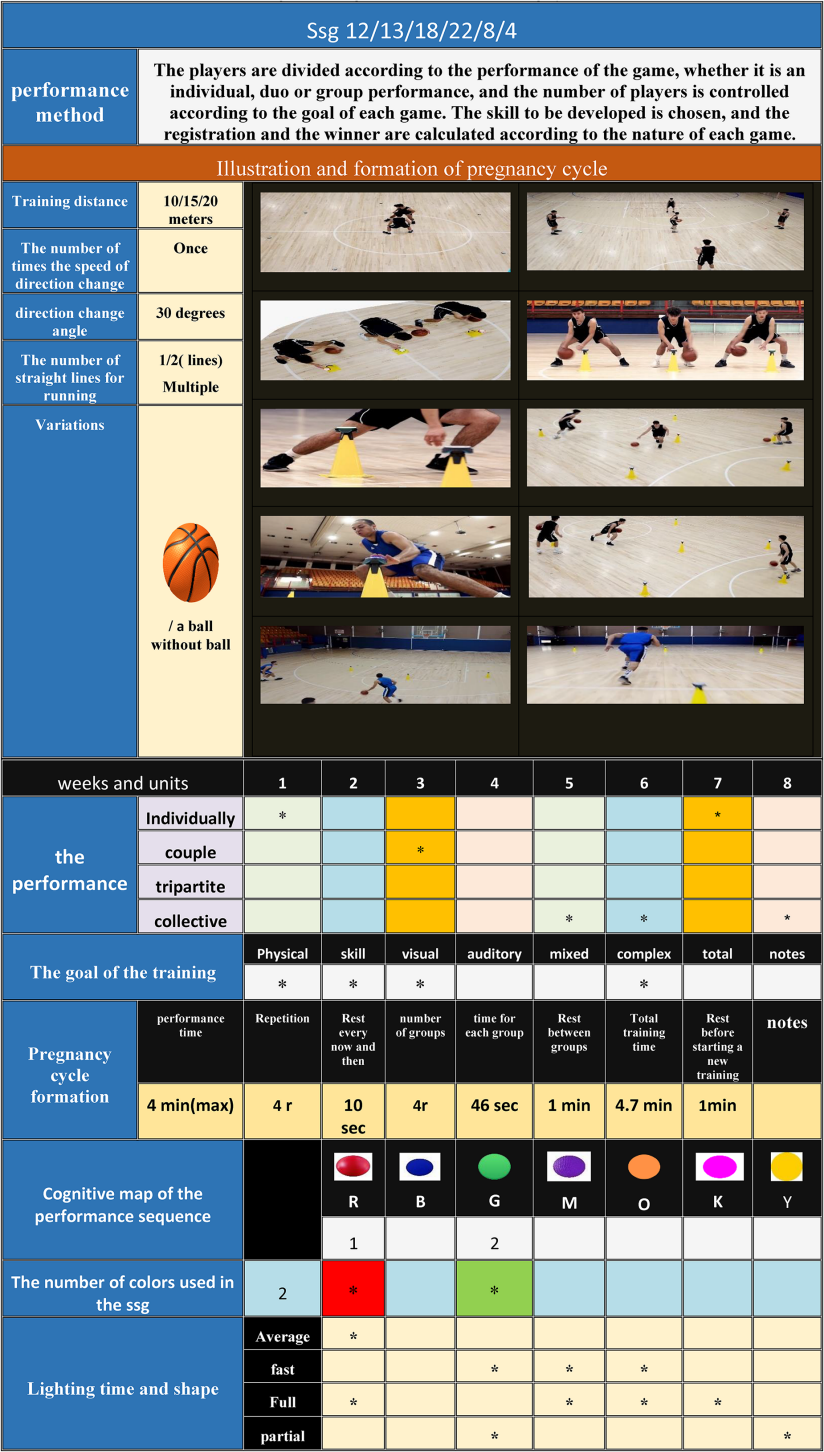

#### 2.3.2. Testing procedures

Before starting the program, a pre-measurement was conducted for all the research variables in the two groups. Firstly, there was a uniform warm-up for 15 min. This involved walking, jumping, and running short distances. All exercises were completed individually and without causing the players to become tired. Secondly, the tests were carried out in a random way to avoid fatigue from one test to another. Thirdly, verbal encouragement was given to motivate the research sample to perform each test to the best of their abilities.In addition, it the harmonic abilities have been evaluated using the Kiko battery (Rhythmization ability -Orientation ability - ability to differentiate - Responsiveness ability - ability to balance) (21–24). (See Appendix A), and basketball skillsThrough skill tests to measure (PASS - dribbling and Throw) (25–29). (See Appendix B), The researchers conducted the pre- testes during the period of 29– 30 March 2022. The researchers took into account the application of tests to all individuals in a unified manner. The proposed training program Using Fitlight was applied for Ten weeks starting on 2 April 2022 and ending on 9 June 2022, and the traditional program applied to the members of the control group. After the completion of the application of the program, telemetry of the two research groups in the period from 11 to 12 June 2022, using the same method that was followed in the pre-measurement and under the same conditions.

#### 2.3.3. Statistical analysis

The Statistical Package for the Social Sciences (SPSS) (IBM SPSS Statistics 26.lnk, Chicago, IL, USA) was used for the statistical analyses. The mean and standard deviation were calculated. A t-test analysis, Cohen's d and the change ratio were applied in this study. The significance level was set at *p* < 0.05.

#### 2.3.4. Results

We presented The results in Tables 3–5, indicating the differences between the experimental and control groups in the variables under consideration.

**Table 3:** Indicates the differences between the averages of pre- and post- measurements of the experimental group in the variables under consideration (*n* = 12).

Variables	Measruing unit	Pre		Post		95% Confidence Interval of the Difference		T	Cohen's d	Sig	Change Ratio
		Mean	SD. Deviation	Mean	SD. Deviation						
						Lower	Upper				
Rhythmization ability	Number	10.17	0.72	5.50	0.52	4.13	5.20	24.82	7.16	0.00	45.92%
Orientation ability	Number	3.42	0.51	7.25	0.87	−4.44	−3.23	12.89	3.72	0.00	111.99%
Ability to differentiate	CM	18.96	0. 20	10.50	0.37	8.20	8.70	59.03	17.04	0.00	44.62%
Responsiveness ability	CM	179.50	3.87	122.58	2.39	54.19	59.64	44.40	12.82	0.00	31.71%
Ability to balance	Number	9.33	0.49	16.42	0.67	−7.58	−6.59	36.70	10.59	0.00	75.99%
PASS	Number	10.75	0.75	18.25	0.62	−8.08	−6.91	49.75	14.36	0.00	69.77%
Dribbling	Sec	11.03	0.19	8.76	0.19	2.11	2.43	36.53	10.55	0.00	20.58%
Throw	Number	4.08	0.67	6.83	0. 39	−3.21	−2.29	10.56	3.05	0.00	67.40%

Statistical significance (Sig.), T- value of t at the significance level of 0.05 = 1.796.

**Table 4:** Indicates the differences between the averages of pre- and post- measurements of the Controlled group in the variables under consideration (*n* = 12).

Variables	Measruing unit	Pre		Post		95% Confidence Interval of the Difference		T	Cohen's d	Sig.	Change Ratio
		Mean	SD. Deviation	Mean	SD. Deviation						
						Lower	Upper				
Rhythmization ability	Number	10.17	0.83	7.00	0.60	2.51	3.82	10.65	3.07	0.00	31.17%
Orientation ability	Number	3.33	0.49	5.17	0.39	−2.08	−1.59	16.32	4.71	0.00	55.26%
Ability to differentiate	CM	18.92	0.20	15.78	0.58	2.74	3.54	17.23	4.97	0.00	16.60%
Responsiveness ability	CM	180.75	4.02	163.42	7.40	13.49	21.18	9.92	2.86	0.00	9.59%
Ability to balance	Number	9.25	0.45	13.25	.45	−4.38	−3.62	22.98	6.63	0.00	43.24%
PASS	Number	10.92	0.79	17.33	1.87	−7.94	−4.90	9.29	2.68	0.00	58.70%
Dribbling	Sec	11.08	0.17	10.18	0.31	.69	1.09	9.65	2.79	0.00	8.12%
Throw	Number	4.08	0.67	5.50	0.52	−2.92	−1.91	9.53	2.75	0.00	34.80%

Statistical significance (Sig.), T- value of t at the significance level of 0.05 = 1.796.

**Table 5:** Indicates the differences between the averages of post measurements of the experimental and controlled groups in the variables under consideration (*n* = 24)

Variables	Measruing unit	Experimental		Controlle		95% Confidence Interval of the Difference		T	Cohen's d	Sig.	Differences in Change Ratio
		Mean	SD. Deviation	Mean	SD. Deviation						
						Lower	Upper				
Rhythmization ability	Number	5.50	0.52	7.00	0.60	−1.98	−1.02	6.51	1.33	0.00	14.75%
Orientation ability	Number	7.25	0.87	5.17	0.39	1.51	2.65	7.60	1.55	0.00	56.73%
Ability to differentiate	CM	10.50	0.37	15.78	0.58	−5.69	−4.87	26.66	5.44	0.00	28.02%
Responsiveness ability	CM	122.58	2.39	163.42	7.40	−45.49	−36.18	18.18	3.71	0.00	22.12%
Ability to balance	Number	16.42	0.67	13.25	0.45	2.68	3.65	13.59	2.77	0.00	32.75%
PASS	Number	18.25	0.62	17.33	1.87	−0.27	2.10	1.61	0.33	0.122	11.07%
Dribbling	Sec	8.76	0.19	10.18	0.31	−1.64	−1.21	13.73	2.80	0.00	12.46%
Throw	Number	6.83	0. 39	5.50	0.52	0.94	1.72	7.09	1.45	0.00	32.60%

Statistical significance (Sig.), T- value of t at the significance level of 0.05 = 2.074.

##### 2.3.4.1. Figures, tables and schemes

**Figure 2:**
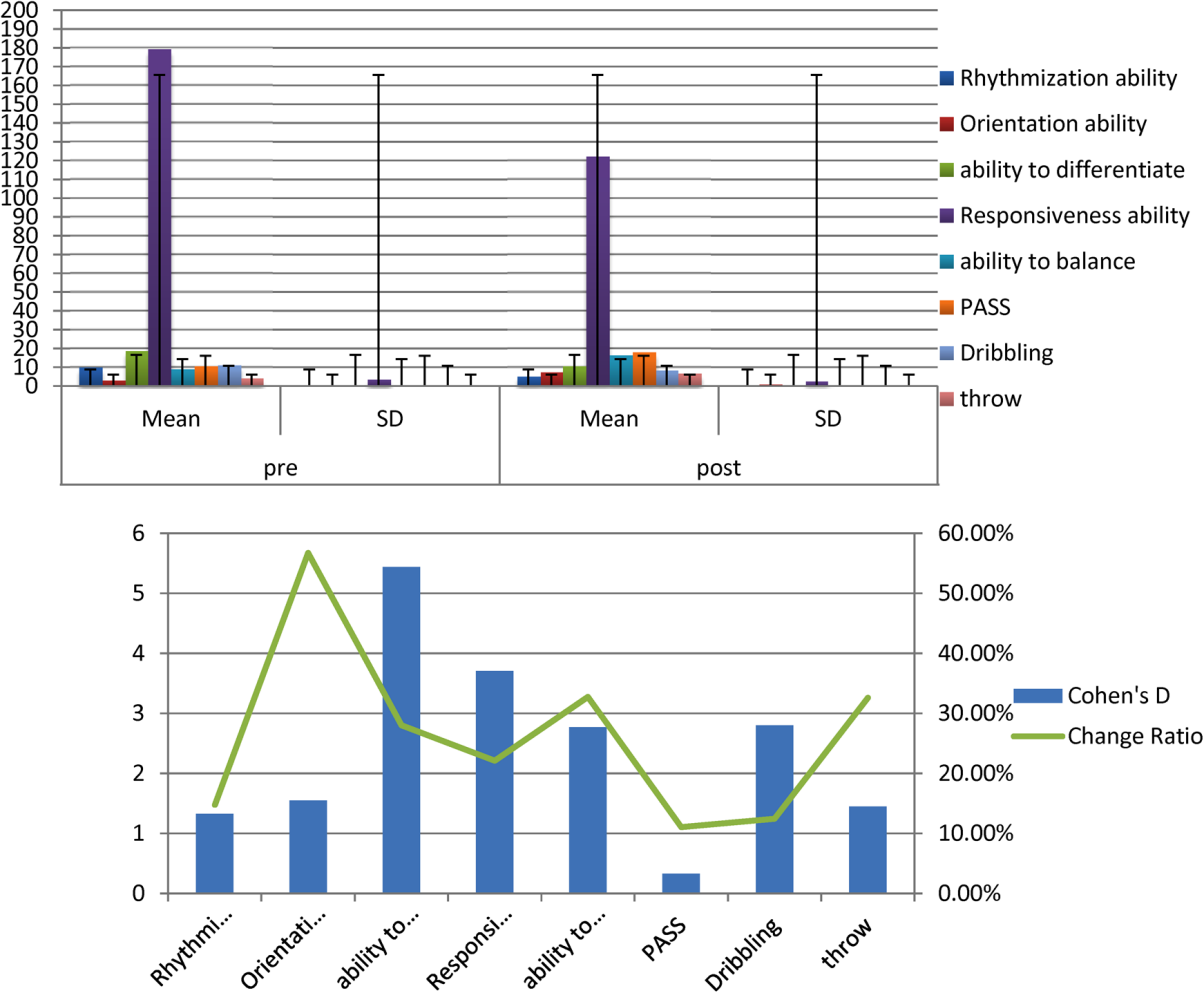
Indication of differences between the averages of pre and post measurements of the experimental group in the variables under consideration.

The average, standard deviation values and Cohen's d of the pre- and post-measurement of the experimental group of all variables examined for players illustrate Figure 2, Table 3.

Table 3 displays the differences between The mean of the pre and post measurements of the experimental group in the variables under discussion, T- value, Cohen's d and the percentage of improvement in the direction of the post measurement.

Table 3 shows the statistical function differences between the pre- and post-measurements of the experimental group in the variables under consideration and is in favor of the dimensional measurement as all the calculated values of T are greater than the tabular value of T at the level of significance (0.05).

**Figure 3:**
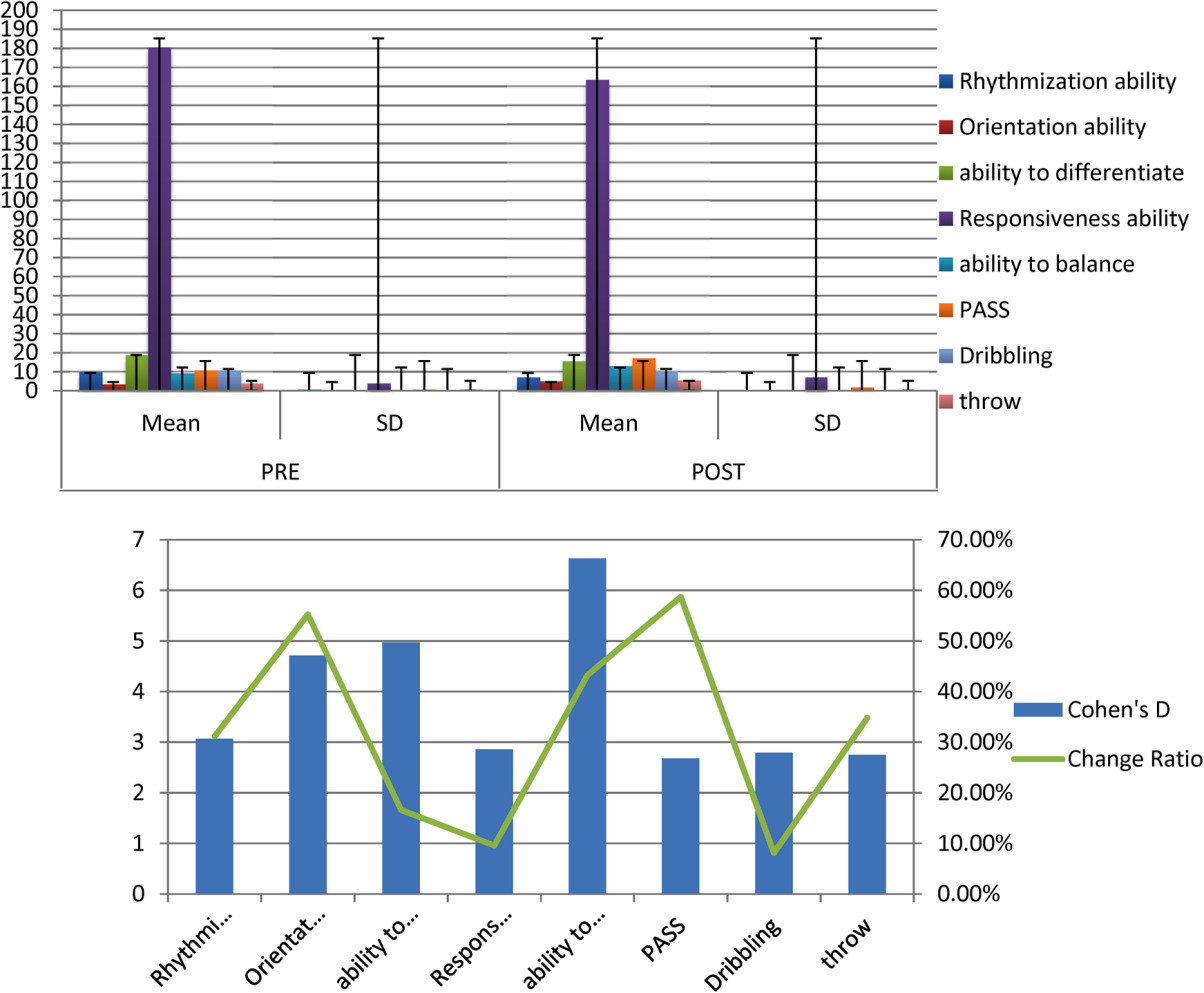
Indication of differences between the averages of pre and post measurements of the controlled group in the variables under consideration.

The average, standard deviation values and Cohen's d of the pre- and post-measurement of the Controlled group of all variables examined for players illustrate Figure 3.

Table 4 displays the differences between The mean of the pre and post measurements of the Controlled group in the variables under discussion, T- value, Cohen's d and the percentage of improvement in the direction of the post measurement.

Table 4 there are statistically significant differences between the pre- and post-measurements of the control group in the variables under consideration. They present in favor of post measurements as all the calculated values of T are greater than the value of T tabular at the level of significance (0.05).

**Figure 4:**
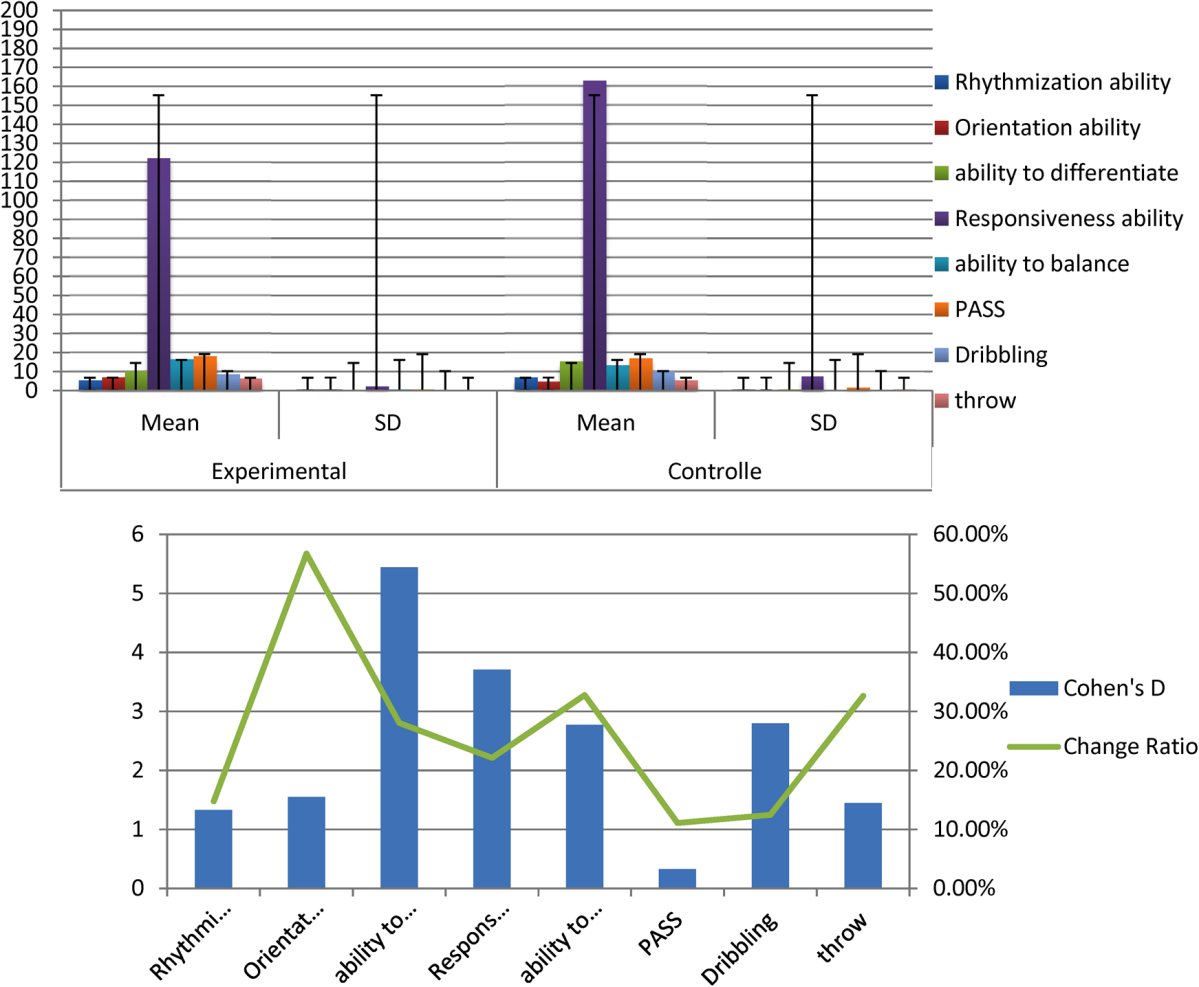
Indication of differences between the averages of post measurements of the experimental and controlled groups in the variables under consideration.

The average, standard deviation values and the T value of post measurements of the experimental and controlled groups of all variables examined for players illustrate Figure 4.

Table 5 there are statistically significant differences between the experimental and control research groups in the variables under consideration. These are in favor of the experimental group as all calculated values of T are greater than the tabular value of T at the significance level 0.05.

### 2.4. Discussion

Table 3 shows the statistical function differences between the pre- and post-measurements of the experimental group in the variables under consideration and is in favor of the dimensional measurement as all the calculated values of T are greater than the tabular value of T at the level of significance (0.05).

Thi shows that there is progress in the harmonic abilities and basic skills with the use of **Small-sided** games, using FITLIGHT. FITLIGHT provided young people with fun and excitement through motor interaction with a new technology and **Small-sided** games. Regarding the muscular and motor performance of basketball skills, FITLIGHT is designed in a way that combines competition, performance and fun, which has played a major role in the development of the level of harmonic and skill abilities (30–32). emphasize the need to train harmonic abilities, particularly amongst young people, where the improvement of skills and performance depends on the development of harmonic abilities (32, 33).

As can be seen from Table 4, there are statistically significant differences between the pre- and post-measurements of the control group in the variables under consideration. They present in favor of telemetry as all the calculated values of T are greater than the value of T tabular at the level of significance (0.05).

The researchers attribute this progress in the harmonic abilities and basic skills to the program followed, which contained physical and skill training. This program increased the level of some harmonic abilities and skill variables. This progress is also due to the regularity of the implementation of the training program among the members of the control group. It is also due to the continuation of practice in addition to the continuous competition between the members of the control group. For young people, providing the best physical and skill performance greatly raised the level of some of thier abilities and basic skills. The researchers also attribute this progress to the role of the trainer in training motor skills by providing a set of graded exercises, which were suitable for the age stage of the trainees.

As can be seen from Table 4, there are statistically significant differences between the experimental and control research groups in the variables under consideration. These are in favor of the experimental group as all calculated values of T are greater than the tabular value of T at the significance level 0.05.

This indicates that the traditional method is insufficient in providing physical effort and motor experiences for young people, when compared to the small games program using FITLIGHT. This is because of its reliance on simple, traditional and often boring movements. It lacks some important elements which are related to good performance, such as the elements of suspense, fun and competition. This contrast the experience which FITLIGHT offers. FITLIGHT provides a fun and exciting environment which aids skill development through the principle of reward without punishment. Therefore, FITLIGHT has a clear practical meaning and offers small games supported by light stimulus. These contain a lot of activities, such as running, running with change of direction, races, jumping movements, partridge, rolling and throwing, These gams promote coordination, helping young people to develop their motor, harmonic and other skills. This increased the desire of the young people to compete and exert effort, thus improving their harmonic and skill abilities. This is consistent with the findings of studies confirmed asserted that small games have a positive and marked effect on control, dribbling, throwing, running, defensive and offensive agility, skills and improving playfulness (22, 33–35).

The researchers attribute this to the positive effect of FITLIGHT, which contains quality guided exercises (18). It also offers diversity in **Small-sided** games and training situations that suit the abilities and habits of the players, attracting attention (36), integrating the movements of the arms and legs and providing light stimulus to develop harmonic abilities. This, alongside the continuous training, led to an improvement in the young players' concentration and accuracy.

This finding is consistent with FG Team's report that FITLIGHT Trainer can help trainers to improve vital aspects of emerging performance, including speed, agility, acceleration capabilities, visual cognitive processing, reaction response, fluidity of movement, physical adaptation, motor skills, cognitive skills, concentration, ambient awareness, vertical lifting ability, multi-directional change and hand-eye coordination. The system also helps trainers track progress in injury recovery (37, 38).

The researchers also believe that the high rate of improvement in the level of harmonic abilities and basic skills was due to the program's inclusion of small games using FITLIGHT. Harmonic exercises were not included in the control group program. These are considered an educational and training alternative that have positive effects on harmonic abilities and basic skills for young players.

This is consistent with findings from Kosel A (24). who states that there is a correlation between harmonic abilities and physical abilities. This means that harmonic abilities are considered the basis for the acquisition of physical abilities and that mastery in motor skills can only been achieved through the comprehensive development of physical and harmonic abilities because skill improves with the improvement of these abilities.

The reason behind these differences can also be attributed to the content of Small-sided games using FITLIGHT. Small-sided training games are increasingly being used asa means of improving the skill and physical fitness levels of team sport athletes (36, 39). The exercises and games were simple so that the young players could easily perform.The factors of suspense, encouragement and competition were important. The integration of light stimulus into the games was also crucial (40). There is an important link between physical motor performance, skills and photo stimulus. The photo stimulus excites the nervous system by sending signals and permanent information to the eye through light stimuli, which has had a positive impact on improving the harmonic abilities and basic skills of basketball players. It also allows them to perform motor duties accurately and quickly. Furthermore, it promotes agility, endurance and high fluidity. The length of time of the execution of motor duties contributed to the development of coordination between the lower and upper limbs, in addition to hand-eye coordination (18). This has helped to develop the harmonic abilities and basic skills of the players, as well as making the basic movements and various motor abilities of these games easier. These include walking, running, jumping, throwing, standing, compatibility, agility, balance and flexibility (41). It also gives players the opportunity to recognize their physical potential, helping them to develop their overall motor growth.

FITLIGHT is a fast and cognitive training system that uses photo stimulus with a variety of training protocols (38). The FITLIGHT system can improve a range of motion, strength, coordination, body comprehension, agility, balance and neurocognitive disorders (30). Millanovic Z. et al. (31) argue that athletic performance includes a visual aspect and a kinetic aspect. Therefore, it is necessary to link the visual aspects using visual stimuli with performance during training and when the visual side develops, the motor aspects also evolve.

The training program used in the research also contained Small-sided games using FITLIGHT which applied exercises that linked physical and skill requirements. The suitability of the size of the physical and skill training loads included in the training units were appropriate. The physical and skill abilities of the players, and the atmosphere of competition with the games and exercises, led to this development (42, 43). It also led to the regularity of young people's participation in training, in which they practiced training that was unfamiliar to them. This tightened the ability of the players to implement training modules apply their skills well. Furthermore, the Small-sided games using FITLIGHT simulated training situations which were similar to competitive situations on the field. They further simulated movements on the field to improve the harmonic abilities and basic skills of basketball players (44, 45).

This is consistent with the results of a study Clemente F. et al. (35), which suggest that Small-sided games are smaller and modified versions of official team sports (45). Furthermore, these games are training exercises which are suitable for players of all abilities and levels (46). They constitute an alternative to traditional fitness and skill training in basketball, showing the importance of these games for basketball training. The researchers therefore believe that the training program using FITLIGHT contributed significantly to the improvement and development of harmonic abilities and basic skills.

## 3. Conclusions

The experimental group achieved remarkable development in all the variables under research. Furthermore, Small-sided games using FITLIGHT played an active and significant role in improving the harmonic abilities and basic skills of the basketball players. This led to significant differences between pre- and post-tests, in favor of telemetry. The experimental group achieved development in all the variables under research. There were significant differences between the pre- and post-tests among the members of the control group, who were subjected to the traditional program. Furthermore, The effect of Small-sided games using FITLIGHT during the units of the experimental group was positive within all the variables under research in the experimental group. This was not the case for the control group, meaning that significant differences exist between the dimensional measurements.

## Data Availability

The original contributions presented in the study are included in the article/Supplementary Material, further inquiries can be directed to the corresponding author/s.

## Appendix A: Compatibility Ability Tests

The researchers conducted personal interviews with a group of experts in the field of basketball to survey their opinions on the harmonic abilities of juniors in basketball. They also conducted a survey of previous research and studies, such as Arslan E. et al. (22), Prätorius B. et al. (23), Glassuer G (21)., Kosel A (24). to identify the most important harmonic abilities in the field of sports, thus identifying and ranking the most important harmonic abilities of basketball juniors. The researchers selected the tests suggested by Kiko (2008) to assess the abilities of basketball juniors, which are as follows: Rhythmization ability: test the number of stability in 20s and the unit of measurement number. Orientation ability: test the number of balls that have been flipped and the unit of measurement of the number. Ability to differentiate: jump test from above a divided box and unit of measurement. Responsiveness ability: test the sliding ball and the unit of measurement. Ability to balance: test the number of rollers passed over in 20 s and the unit of measurement.

## Appendix B: Skillful Tests

The researchers conducted a survey of scientific references, research and previous studies in the field of basketball. Literature by scholars, such as Hagedorn G. et al. (25), Schrittwieser M. et al. (26), O'Grady C. et al. (27), Reina M. et al. (28), Versic S. et al. (29), Weineck J. & Haas H (32)., Vencúrik T. et al. (47), Poureghbali S. et al. (12), were drawn upon. The following tests were selected: Chest pas – Dribbling - Free throw.

## Appendix C: Training unit model

This appendix contains a training model with Small-Sided games using FITLIGHT.
